# A 12-month longitudinal naturalistic follow-up of cariprazine in schizophrenia

**DOI:** 10.3389/fpsyt.2024.1382013

**Published:** 2024-05-21

**Authors:** Claudia Carmassi, Valerio Dell’Oste, Sara Fantasia, Andrea Bordacchini, Carlo Antonio Bertelloni, Pietro Scarpellini, Virginia Pedrinelli

**Affiliations:** ^1^ Department of Clinical and Experimental Medicine, University of Pisa, Pisa, Italy; ^2^ Department of Biotechnology, Chemistry and Pharmacy, University of Siena, Siena, Italy; ^3^ Unità Funzionale Complessa Salute Mentale Adulti Zona Valdinievole, Azienda USL Toscana Centro, Montecatini Terme, Italy; ^4^ Unità Funzionale Salute Mentale Adulti Zona Apuana, Azienda USL Toscana Nord Ovest, Massa, Italy

**Keywords:** cariprazine, schizophrenia, psychosis, extrapyramidal symptoms, side effects

## Abstract

**Background:**

Cariprazine, a third-generation antipsychotic (TGAs), has demonstrated efficacy in the treatment of schizophrenia with good tolerability profile. Actual real-world literature data are lacking, particularly when exploring its efficacy in the long term. The present study examined the effects of cariprazine treatment on specific psychopathological domains with a particular focus on outcomes and side effects in real-life experience, after a long-term treatment.

**Methods:**

The present 12-month longitudinal naturalistic study included a sample of subjects with a DSM-5-TR diagnosis of schizophrenia, recruited in the outpatients’ psychiatric services of university and community hospitals in Italy, naturally treated with cariprazine. The assessments included: a sociodemographic data sheet, the Structured Clinical Interview for the DSM-5 (SCID-5), the Positive and Negative Symptom Scale (PANSS) and the St. Hans Rating Scale (SHRS). The PANSS was also administered after 6 (T1) and 12 (T2) months of treatment with cariprazine while the SHRS at T1.

**Results:**

The total sample consisted of 31 patients, 15 males and 16 females. A significant decrease of the PANSS’ subscales, Marder factors and total mean scores emerged at both T1 and T2 with respect to T0. Extrapyramidal symptoms occurred in a minority of patients and in mild or mild/moderate forms: no patient showed moderate forms of psychic/motor akathisia or dystonia, three subjects showed moderate parkinsonism.

**Conclusions:**

This study confirms a good efficacy profile of cariprazine in both positive and negative symptoms in patients with Schizophrenia, combined with a good tolerability profile in extrapyramidal symptoms.

## 1 Introduction

Schizophrenia is a psychiatric syndrome encompassing several symptomatologic domains that have to be considered for its accurate clinical characterization (positive symptoms, negative symptoms, cognitive impairment, mood and anxiety symptoms, etc.) (1). Its course and outcomes are heterogeneous and the prognosis often uncertain (2) so that schizophrenia is considered one of the most disabling and economically catastrophic illnesses and has been listed as one of the top 10 diseases (psychiatric and non-psychiatric) contributing to the global burden of disease (3, 4).

The treatment of schizophrenia requires a multidisciplinary approach and from a pharmacological perspective, antipsychotics are the first choice. In clinical trials, they have been shown to be effective in treating positive symptoms but most of the drug classes tested have not shown clinically significant effects on negative symptoms, including blunted affect, avolition, alogia, anhedonia, and asociality (2, 5, 6). Notably, first-generation antipsychotics (FGAs) may exacerbate negative and cognitive symptoms (7). However, most recent data appear to show improvement in negative symptoms when patients are treated with cariprazine (8).

Cariprazine is a third-generation antipsychotic approved by both the EMA and the FDA for the treatment of schizophrenia (9, 10). It exerts its therapeutic effects through a combination of partial agonist activity at the dopamine D3, D2, and serotonin 5-HT1A receptors and antagonist activity at the serotonin 5-HT2B, 5-HT2A, and histamine H1 receptors. It has two main metabolites, desmethyl-cariprazine and didesmethyl-cariprazine, which have similar receptor binding and functional activity profiles *in vitro* as the original drug (11, 12). Cariprazine safety and tolerability data based on clinical trials have been confirmed in real-life patients (13, 14). In addition, cariprazine has been shown to be effective for a variety of symptom profiles, from acute psychotic symptoms to addiction to negative and cognitive symptoms (13, 15). However, the actual real-life literature data are insufficient and mostly derived from case reports, which are anecdotal and inherently biased.

Given these premises, the aim of this naturalistic longitudinal study is to investigate the treatment effects of cariprazine in individuals diagnosed with schizophrenia, focusing on its effects on specific psychopathological domains and its side effects, in real-life experience.

## 2 Materials and methods

### 2.1 Study sample

A total sample of 31 subjects with a DSM-5-TR diagnosis of Schizophrenia, was consecutively recruited at the adult outpatient psychiatric services of the Psychiatric Clinic of a Major University Hospital in central Italy (Azienda Ospedaliero-Universitaria Pisa, AOUP, Pisa, Italy) between January 2022 and January 2023, and started on cariprazine treatment.

Inclusion criteria were age over 18 years old at the time of enrollment in the study and a DSM-5-TR diagnosis of Schizophrenia. Exclusion criteria comprised poor knowledge of Italian language or other limits to verbal communication and a concomitant diagnosis of cognitive impairment, seizures or other neurological disorders and a history of traumatic brain injuries.

All participants provided a written informed consent upon the opportunity to ask questions, after being clearly informed about the study. All data were collected anonymously.

The study was conducted in accordance with the Declaration of Helsinki and was approved by the Ethics Committee of the Area Vasta Nord Ovest Toscana (Italy).

### 2.2 Assessment instruments

Trained psychiatrists or residents in psychiatry of the Psychiatric Clinic of the University of Pisa, Italy, assessed all subjects enrolled. The assessments included a datasheet for socio-demographic characteristics, including gender, age, marital status, employment status, occupational role, as well as clinical data regarding physical and psychiatric comorbidities, psychopharmacological treatment characteristics and number of prior hospitalizations. The whole sample was investigated by means of psychometric instruments, including: the Structured Clinical Interview for Mental Disorders (SCID-5), for the diagnosis of Schizophrenia and any other mental disorders comorbidities; the Positive and Negative Symptom Scale (PANSS), to measure the psychotic spectrum’s symptom severity; the St. Hans Rating Scale (SHRS) for the evaluation of extrapyramidal drug-induced side effects. Patients were evaluated at baseline before starting the treatment with cariprazine (T0) with all assessment instruments but the SHRS. This latter was performed after 6 (T1) months of cariprazine treatment. The PANSS was also administered after 6 (T1) and 12 (T2) months of treatment with cariprazine.

The Positive and Negative Symptom Scale (PANSS) is a scale including 30 items to assess the severity of psychopathology in adults with schizophrenia. The patient is given a score from 1 to 7 for each item, with a minimum score of 30 and a maximum score of 210. The scale refers to Crow’s two-dimensional model and adds a dimension called “general psychopathology” so that, in addition to the total score, the scores of three subscales can be obtained: positive symptoms (7 items), negative symptoms (7 items), and general psychopathology (16 items) (16). Further factor analytic studies have been performed to investigate whether the thirty symptoms cluster into specific dimensions might underlie distinct processes in schizophrenia, with most of them supporting a five-factor structure explaining from 51 to 72.3% of the variance (17). These five factors (named Marder PANSS factors) typically include Positive, Negative, Cognitive/Disorganization, Depression/Anxiety, and Excitability/Hostility dimensions (17, 18).

The St. Hans Rating Scale (SHRS) is a multidimensional rating scale developed in 1970 and widely used to assess neuroleptic-induced extrapyramidal syndrome. It consists of 4 domini: hyperkinesia passive and active (assessed globally and for individual body regions), parkinsonism (including facial expression, bradykinesia, tremor posture, arm swing, gait, rigidity, and salivation), akathisia, and dystonia. Each item is assigned a score from zero (absent) to 6 (severe) (19).

### 2.3 Statistical analysis

All statistical analyses were performed using the Statistical Package for Social Science, version 22.0 (SPSS Inc.). Continuous variables were reported as mean ± standard deviation (SD), whereas categorical variables were reported as percentages. All tests were two-tailed and a p value <.05 was considered statistically significant.

Paired t-test were used to compare PANSS total score, positive, negative and general psychopathology scales, as well as the five Marder PANSS factors (Positive symptoms, Negative symptoms, Disorganized thought, Hostility/Excitement, Anxiety/Depression) between baseline (T0) and 6-months follow-up (T1) and between T0 and 12-months follow-up (T2)in the total sample. Furthermore, non-parametric Wilcoxon test was used to compare each PANSS’ scale item between T0 and T1 and between T0 e T2 in the total sample.

## 3 Results

The total sample included 31 subjects with a diagnosis of Schizophrenia, 15 (48.3%) males and 16 (51.7%) females, with a mean age of 41.9 ± 10.7 years (40.9 ± 10.9 and 42 ± 11.4 years in males and females, respectively). Socio-demographic and clinical characteristics in the overall sample are summarized in **Table 1**. Regarding socio-demographic characteristics: 7 (23.3%) subjects were married/cohabiting, 14 (45.2%) were employed and 14 (45.2%) had a high school degree. Examining in further detail the clinical characteristics of the sample, 7 (22.6%) subjects had a concomitant diagnosis of a current anxiety disorder, 3 (9.7%) of a current Obsessive Compulsive Disorder and, approximately one third (11, 35.5%) of participants reported a lifetime substance or alcohol use disorder, among which 4 (12.9%) subjects a current alcohol use disorder, 3 (9.7%) a current cannabinoid use disorder, 8 (25.8%) a current stimulant use disorder and 3 (9.7%) a current opioid use disorder. No statistically significant gender differences emerged when comparing socio-demographic and clinical variables (see **Table 1**).

**Table 1:** Sociodemographic and clinical characteristics in the total sample (N=31).

	Total sample N (%)	Males N (%)	Females N (%)	p*
*Married/cohabiting*	7 (23.3)	3 (21.4)	4 (25.0)	1.000
*Employed*	14 (45.2)	7 (46.7)	7 (43.8)	1.000
*High school degree*	14 (45.2)	8 (53.3)	6 (37.5)	.600
Current Medical comorbilities				
*Obesity*	6 (19.4)	3 (20.0)	3 (18.8)	1.000
*Dyslipidemia*	6 (19.4)	4 (26.7)	2 (12.5)	.394
*Hypertension*	5 (16.1)	3 (20.0)	2 (12.5)	.654
*Diabetes Mellitus*	2 (6.5)	1 (6.7)	1 (6.3)	1.000
Current psychiatric comorbilities				
*Anxiety disorder*	7 (22.6)	3 (20.0)	4 (25.0)	1.000
*Obsessive-compulsive disorder*	3 (9.7)	2 (13.3)	1 (6.3)	.600
*Alcohol or substance use disorder*	11 (35.5)	5 (33.3)	6 (37.5)	1.000
*Alcohol use disorder*	4 (12.9)	3 (20.0)	1 (6.3)	.333
*Cannabinoid use disorder*	3 (9.7)	2 (13.3)	1 (6.3)	.600
*Stimulant use disorder*	8 (25.8)	3 (20.0)	5 (31.3)	.685
*Opioid use disorder*	3 (9.7)	0 (0)	3 (18.8)	.226
Cariprazine treatment duration				
*6 months*	31 (100)	15 (100)	16 (100)	Not applicable
*12 months*	16 (51.6)	8 (53.4)	7 (50.0)	Not applicable
*18 months*	7 (22.6)	4 (26.7)	3 (18.8)	Not applicable
*Hospitalizations before starting cariprazine treatment*	15 (48.4)	8 (53.3)	7 (43.8)	.862
*Hospitalizations during cariprazine treatment*	4 (12.9)	2 (13.3)	2 (12.5)	1.000

*comparison between males and females.

The mean cariprazine daily dose in the total sample was 4.1 ± 1.4 mg. A total of 31 (100%) patients had a 6-months treatment duration with cariprazine, whereas data on cariprazine treatment at T2 are available for 29 (93,55%) patients. For what concern cariprazine introduction at T0: 2 (6.5%) patients had not received any antipsychotic; 9 (29%) patients were switched from previous antipsychotic to cariprazine, 20 (64.5%) patients received cariprazine in add-on treatment to previous antipsychotics, (10 quetiapine, 5 chlorpromazine, 5 levomepromazine), these latters being subsequently remodeled according to each patient’s clinical course.

Results also showed that 4 (12.9%) patients reported at least one hospital admission for relapses during cariprazine treatment, while 15 (48.4%) reported at least one hospital admission before starting cariprazine treatment. In more details, one patient was admitted after about a month of cariprazine treatment (at a dosage of 3 mg/day) and was discharged with cariprazine at 3 mg/day in add-on to chlorpromazine 50 mg/day; two patients were admitted after discontinuation of therapy about 3 months after T0 and they were discharged with cariprazine therapy at the previous dose, with clinical benefit lasting to date. Finally, a patient was admitted after treatment discontinuation about 4 months after initiation, was discharged on cariprazine at 4.5 mg/day (same previous dose) associated with sertraline and benzodiazepines (comorbidity with Obsessive Compulsive Disorder).

During the study, two subjects dropped out and did not reach the 12-month follow-up after starting the treatment with cariprazine. Reasons for drop-out were related to the fact these patients moved in another town and were no longer followed at the psychiatric services of the centers involved in the present study.

**Figure 1:**
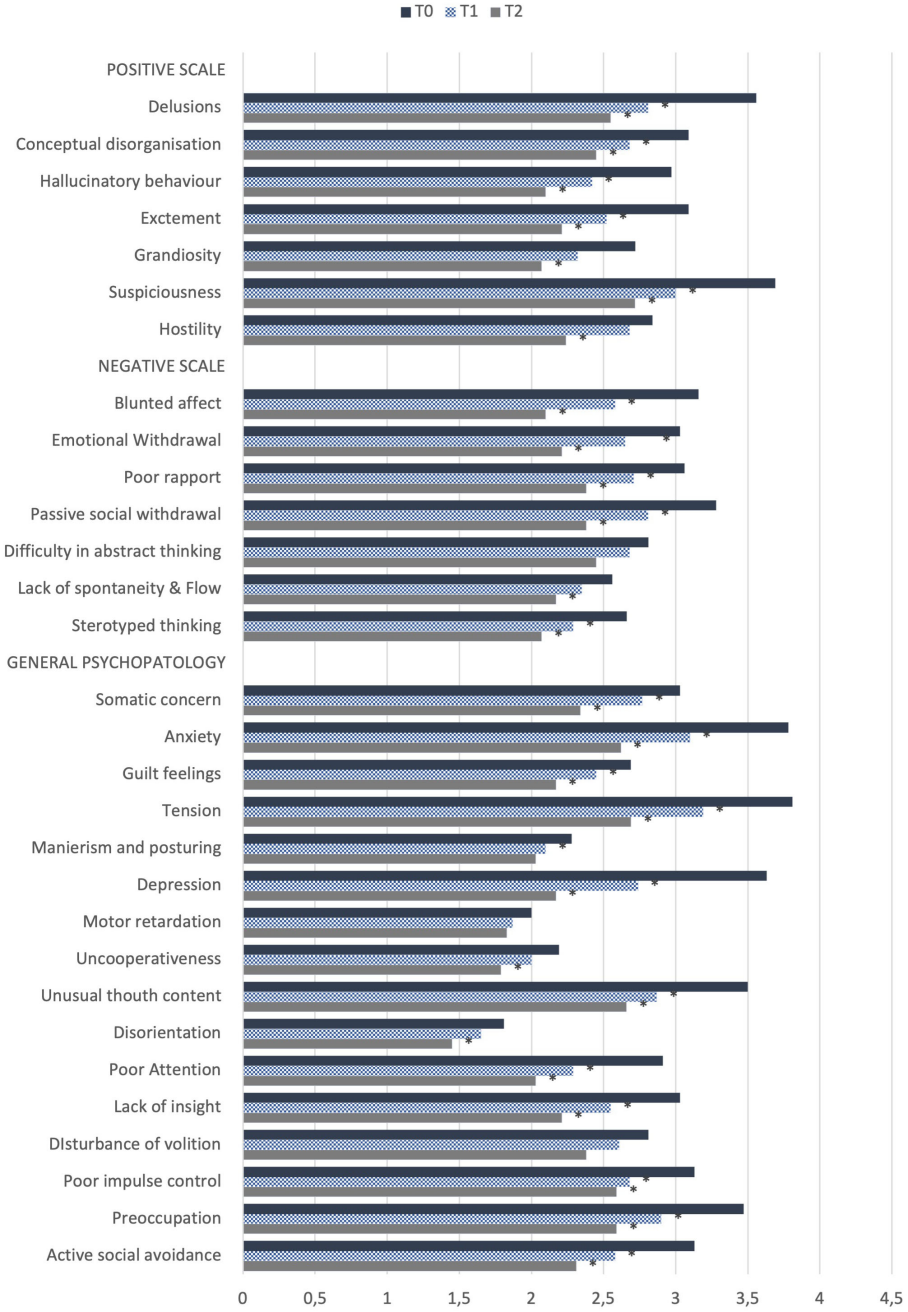
Comparison of PANSS scale single items between T0 (baseline) and T1 (6-months follow-up) in the total sample (N=31) and between T0 and T2 (12-month follow-up) in the total sample (N=29). *p<.05.

When comparing both the PANSS’ subscales and total mean scores between T0 and T1, interesting results emerged (see **Table 2**). An overall significant reduction in the PANSS’ scale scores in the total sample was shown, with a decrease of both the PANSS’ subscales and total mean scores. Focusing on the single items of the PANSS’ subscales between T0 and T1 in the total sample, statistically significant decrease in the PANSS’ *Positive Scale* items scores (delusions, conceptual disorganization, hallucinatory behavior, excitement, suspiciousness), as well as of negative symptoms was reported, with significant lower scores in PANSS’ negative scale items scores: blunted affect, emotional withdrawal, poor rapport, passive social withdrawal and stereotyped thinking. An improvement of general psychopathological symptoms between T0 and T1 was reported too, with a significant decrease in the items regarding somatic concern, anxiety, guilt feelings, tension, mannerism and posturing, depression, unusual thought content, poor attention, lack of insight, poor impulse control, preoccupation and active social avoidance (p<.05, see **Figure 1**). Interestingly, focusing on the Marder PANSS’ factor scores between T0 and T1 in the overall sample, a significant decrease among each of these factors (positive symptoms, disorganized thoughts, negative symptoms, hostility/excitement, anxiety/depression) was shown (See **Table 2**). Finally, at T2 a statistically significant decrease in the PANSS’ total and single subscales scores emerged with respect to T0 (p<.001). Similarly, results from the comparison of Marder PANSS’ factor scores in between these two assessment times showed a substantial improvement in symptoms with a significant decrease for the Positive (p<.001) and Negative (p<.001) factors, as well as for Disorganized thoughts (p<.001), Uncontrolled Hostility excitement (p<.001) and Anxiety Depression (p<.001) ones (see **Table 2**). Further, the comparison of PANSS single items scores between T0 and T2 demonstrated a significant reduction of all items in the *Positive Scale*, as well as in the *Negative Scale* with the exception of difficulties in abstract thinking and of all items in the *General Psychopathology Scale*, except for motor retardation, mannerism and posturing and disturbance of volition (p<.05, see **Figure 1**).

**Table 2:** PANSS scale scores’ comparison between T0 (baseline) and T1 (6-month follow-up) in the total sample (N=31) and between T0 and T2 (12-month follow-up) in the total sample (N=29).

PANSS scores		Total sample mean ± SD	p	PANSS scores	Total sample mean ± SD	p
*Positive scale*	T0	21.7 ± 5.7	.001	T0	21.7 ± 5.9	<.001
	T1	18.42 ± 5.9		T2	16.4 ± 5.9	
*Negative scale*	T0	20.8 ± 7.8	.002	T0	19.9 ± 7.5	<.001
	T1	18.0 ± 7.8		T2	15.7 ± 6.5	
*General psychopatology scale*	T0	47.3 ± 10.5	<.001	T0	47.4 ± 10.8	<.001
	T1	40.3 ± 11.4		T2	35.8 ± 9.5	
*Total score*	T0	89.7 ± 19.0	<.001	T0	89.2 ± 19.4	<.001
	T1	76.8 ± 22.2		T2	67.9 ± 19.0	
*Marder PANSS’ positive symptoms factor*	T0	25.1 ± 6.4	.<001	T0	25.1 ± 6.7	<.001
	T1	21 ± 6.5		T2	18.7 ± 5.7	
*Marder PANSS’ negative symptoms factor*	T0	20.2 ± 6.9	.001	T0	19.7 ± 6.6	<.001
	T1	17.5 ± 7.1		T2	15.3 ± 5.7	
*Marder PANSS’ disorganized though factor*	T0	19.1 ± 5.6	.001	T0	18.8 ± 5.7	<.001
	T1	16.9 ± 5.9		T2	15.3 ± 5.7	
*Marder PANSS’ uncontrolled hostility/excitement factor*	T0	11.2 ± 3.5	.015	T0	11.4 ± 3.7	<.001
	T1	9.9 ± 3		T2	8.8 ± 2.9	
*Marder PANSS’ anxiety/depression factor*	T0	13.9 ± 3.7	.<.001	T0	13.9 ± 3.8	<.001
	T1	11.5 ± 3.8		T2	9.6 ± 3.4	

Concerning extrapyramidal drug-induced side effects, assessed by means of the St Hans scale scores in the total sample at T1 (6-month follow-up, see **Table 3**), akathisia emerged in mild form in 4 (12.9%) patients and in mild/moderate form in 2 (6.5%) patients. Dystonia occurred in mild form in 1 (3.2%) patient and in mild/moderate form in 3 (9.7%) patients. Furthermore, parkinsonism developed in the mild form in 4 (12.9%) subjects, in the mild/moderate form in 2 patients (6.5% of the total sample) and in the moderate form in 3 patients (9.7% of the total sample).

**Table 3:** St Hans scale items' endorsement at T1 (6-months follow-up) in the total sample (N=31).

St Hans items	Total sample N (%)
Psychic Akathisia	
Absent/Dubious	25 (80.6)
Mild	4 (12.9)
Mild/Moderate	2 (6.5)
Moderate	0 (0)
Motor Akathisia	
Absent/Dubious	26 (83.9)
Mild	4 (12.9)
Mild/Moderate	1 (3.2)
Moderate	0 (0)
Dystonia	
Absent/Dubious	27 (87.1)
Mild	1 (3.2)
Mild/Moderate	3 (9.7)
Moderate	0 (0)
Parkinsonism	
Absent/Dubious	22 (70.9)
Mild	4 (12.9)
Mild/Moderate	2 (6.5)
Moderate	3 (9.7)

## 4 Discussion

To the best of our knowledge, this is the first naturalistic 12-month longitudinal study examining the efficacy and tolerability of cariprazine treatment in real-life patients affected by schizophrenia. Results showed a statistically significant improvement in the all PANSS’ domains scores in the total sample at T1 (6-months follow-up). Specifically, a significant improvement emerged in all Positive Symptoms assessed except Hostility and Grandiosity, and in all Negative Symptoms assessed, except Difficulty in Abstract Thinking and Lack of Spontaneity and Flow. Cariprazine also appeared to have a broad spectrum of action in general psychopathology, where it leaded to improvement in somatic concern, anxiety, guilt feelings, tension, mannerism and posturing depression, unusual thought content, poor attention, lack of insight, poor impulse control, preoccupation and active social avoidance. The data also showed a progressive and significant reduction in both the PANSS’ total and single subscales scores, except for difficult in abstract thinking, motor retardation and disturbance of volition. Our data also showed that reduction in the Grandiosity, Hostility and Lack of Spontaneity and Flow subscales became significant at the 12-month follow-up (T2) compared with the baseline, which was coinciding with the start of the treatment with cariprazine. Similar results emerged when comparing the PANSS-derived Marder factors across assessment time points of the study, with significant symptoms’ improvement evidenced by the significant reduction in scores, both between T0 and T1 (6-month follow-up) and between T0 and T2 (12-month follow-up) for each of the factors (positive symptoms, disorganized thoughts, negative symptoms, hostility/excitement, anxiety/depression). These results are consistent with data from the literature showing that cariprazine appear to have a good efficacy profile in a wide range of symptoms of schizophrenia (8, 13, 14, 20–26). Further, a recent study provided support for the notion that cariprazine in high dosages might be a well-tolerated and effective treatment option for acute patients on the schizophrenia spectrum in real-life as well (27). In the present study, in fact, cariprazine proved to be effective controlling positive symptoms both the mid and long-term follow-up. In this perspective, a long-term multi-country, double-blind, placebo-controlled study conducted by Durgam et al. (28), showed that patients treated with cariprazine had a significantly longer time to relapse than those treated with placebo, with relapse occurring in only 24.8% of cariprazine-treated patients versus 47.5% of placebo-treated patients during the open-label 72-week follow-up treatment. Further, a multicenter, open-label, flexible-dose study to evaluate the safety and tolerability of cariprazine in patients with schizophrenia for 53 weeks concluded that cariprazine treatment up to 9 mg/d was generally safe and well tolerated in patients with schizophrenia over a long-term period (29). Moreover, in line with some case studies reporting the efficacy of cariprazine in mitigating the hostility in acute patients with psychosis (21, 30), our results confirmed this trend in the sample also in the long-term follow-up, with an improvement shown at the 6 month-follow-up by the reduction of the PANSS Hostility item and more pronounced at the 12-month follow-up, showing the possible effect of the drug in the mid and long-term treatment with cariprazine. Recently, possible pro-cognitive and anti-hostility effects of cariprazine have been also reported in the treatment of resistant schizophrenia (31). Additionally, the chronicity of this disorder also requires a long-term view, which should always contemplate the burden of treatment on negative and cognitive symptoms as well and improvement in this psychopathological profile was evident in the whole sample both after 6 and 12 months of treatment.

In addition, our study also investigated the occurrence of extrapyramidal effects during cariprazine treatment in the sample at the 6-month follow-up. The data from our study are consistent with those already available in the literature (21, 22, 24, 25, 32, 33), substantially showing good tolerability of cariprazine, with extrapyramidal symptoms occurred in a minority of patients and in mild or mild or moderate forms: no patient showed moderate forms of psychic/motor akathisia or dystonia and only three subjects showed moderate parkinsonism. A lower prevalence of akathisia has been reported in cariprazine treatment with respect to other antipsychotic treatments (32).

The distress caused by antipsychotic-induced akathisia can be significant and impact treatment adherence and long-term patient outcomes. Akathisia was the most commonly observed treatment-emergent adverse event with cariprazine, with a mild to moderate in severity (34), in line with our results. Akathisia rarely led to treatment discontinuation because most patients were able to effectively manage akathisia while continuing treatment with cariprazine. Overall, these findings suggest that cariprazine causes fewer cases of akathisia and that most patients can effectively manage this side effect while continuing their treatment with cariprazine (34).

The lower incidence of extrapyramidal effects and efficacy on positive and negative symptoms of schizophrenia observed in the present study may be attributable to the unique pharmacokinetic properties of the drug. Indeed, cariprazine is a D2 and D3 partial agonist, 5HT2b receptor antagonist and 5HT1a receptor partial agonist. Positive symptoms’ control would be attributable to the partial D2 antagonism at mesolimbic level, balanced by 5HT2a receptor antagonism. Unlike total D2 antagonists, which can result in iatrogenic parkinsonism via total D2 blockade in the nigrostriatal pathway and worsening of negative symptoms by decreasing dopaminergic tone at cortical level, partial agonists such as cariprazine allow normalization of dopaminergic stimulation in the mesolimbic system, reducing positive symptoms without grossly impacting the nigrostriatal system; in addition, partial blockade of the 5HT2b receptor allows improved dopaminergic tone in the prefontal cortex, leading to better control of negative symptoms (35).

The peculiar feature of the high-affinity partial D3 agonism shown by cariprazine, may also contribute to improve negative symptoms and cognitive deficits associated with schizophrenia and related disorders. Indeed, determining a partial blockade of D3 receptors in the ventral tegmental area, cariprazine causes an increase in dopaminergic tone in the prefrontal cortex, a peculiar feature of the drug that allows an additional pathway to control these symptoms (36).

Cariprazine also has a relatively high affinity for 5HT1A receptors that has been associated with improved negative symptoms and cognitive deficits (26, 37). The 5HT1A receptor is a subtype of serotonin receptor widely distributed throughout the brain and involved in several physiological processes, including mood, cognition and stress response (26, 38–41). Activation of the 5HT1A receptor has been shown to have potential models antidepressant and anxiolytic effects and pro-cognitive effects in animal and clinical studies. Similarly, our results showed that long-term treatment with cariprazine was also associated with a significant improvement in a wide spectrum of general psychopathology symptoms, including depressive, anxiety, hostility and excitement ones.

This study has several strengths: first, the naturalistic longitudinal study design. In addition, the efficacy of the therapy was assessed not only by the overall improvement in the psychopathological profile, but also by the effects on each symptom of PANSS scale. However, when discussing the results obtained, it is also necessary to point out possible limitations such as the small sample size, despite the naturalistic real-life design of the study allows to shed light on the potential usefulness of this medication. Expanding the sample size also including participants from a broader range of geographic locations and diverse backgrounds, in fact, may enhance the robustness and applicability of the study findings, helping to understand the variability in response to cariprazine. Further, as the assessment of extrapyramidal symptoms among subjects recruited was carried out only at T1 (6-month follow-up) providing only an initial insight into these side effects associated with cariprazine treatment, the results of our study should be interpreted in the context of this limitation.

In our study, cariprazine seems to confirm its efficacy profile in both negative and positive symptoms, combined with good tolerability in real-life patients with Schizophrenia. Further longitudinal study on cariprazine, particularly orientated to gender-specific tailored medicine, are needed.

## Data availability statement

The raw data supporting the conclusions of this article will be made available by the authors, without undue reservation.

## Ethics statement

The studies involving humans were approved by Ethics Committee of the Area Vasta Nord Ovest Toscana (Italy). The studies were conducted in accordance with the local legislation and institutional requirements. The participants provided their written informed consent to participate in this study.

## Author contributions

CC: Writing – review & editing, Writing – original draft, Supervision, Methodology, Investigation, Conceptualization. VD: Writing – review & editing, Writing – original draft, Supervision, Methodology, Investigation, Formal analysis, Data curation, Conceptualization. SF: Writing – review & editing, Writing – original draft, Investigation. AB: Writing – review & editing, Writing – original draft. CB: Writing – original draft, Investigation, Formal analysis, Data curation. PS: Writing – original draft, Supervision, Methodology. VP: Writing – review & editing, Writing – original draft, Supervision, Methodology, Investigation, Data curation, Conceptualization.
